# A temperature sensitive *Mycobacterium paragordonae* induces enhanced protective immune responses against mycobacterial infections in the mouse model

**DOI:** 10.1038/s41598-017-15458-7

**Published:** 2017-11-09

**Authors:** Byoung-Jun Kim, Bo-Ram Kim, Yoon-Hoh Kook, Bum-Joon Kim

**Affiliations:** Department of Microbiology and Immunology, Biomedical Sciences, Liver Research Institute and Cancer Research Institute, College of Medicine, Seoul National University, Seoul, Korea

## Abstract

Recently, we introduced a temperature sensitive *Mycobacterium* spp*., Mycobacterium paragordonae* (Mpg). Here, we checked its potential as a candidate for live vaccination against *Mycobacterium tuberculosis* and *Mycobacterium abscessus*. Intravenous infections of mice with Mpg led to lower colony forming units (CFUs) compared to infection with BCG, suggesting its usefulness as a live vaccine. The analyses of immune responses indicated that the highly protective immunity elicited by Mpg was dependent on effective dendritic maturation, shift of cytokine patterns and antibody production toward a Th1 phenotype, and enhanced cytotoxic T cell response. Compared to BCG, Mpg showed a more effective protective immune response in the vaccinated mice against challenges with 2 different mycobacterial strains, *M. tuberculosis* H37Ra or *M. abscessus* Asan 50594. Our data suggest that a temperature sensitive Mpg may be a potentially powerful candidate vaccine strain to induce enhanced protective immune responses against *M. tuberculosis* and *M. abscessus*.

## Introduction

*Mycobacterium tuberculosis* is a leading pathogen causing global mortality approximately 9 million infections and 1.5 million deaths each year^1^. Furthermore, co-infection with HIV and the emergence of multiple drug-resistant strains of *M. tuberculosis* (Mtb) make the control of tuberculosis more difficult^2,3^. The only vaccine currently used for the protection against Mtb infection is the live attenuated variant of *M. bovis*, known as Bacille Calmette-Guérin, or BCG^4^. It has been reported that the BCG vaccine has low or inconsistent efficacy in preventing Mtb infections, and BCG can cause disseminated disease in immunocompromised individuals such as HIV-positive infants^5–7^. Therefore, there is a continuing effort to develop new anti-tuberculosis vaccines that are safer and more effective than BCG^8–11^.

*Mycobacterium abscessus* complex is a rapidly growing *Mycobacterium* that is responsible for a wide spectrum of infections in humans^12,13^. Infections by *M. abscessus* (Mab) complex strains lead to a higher fatality rate than those seen with other rapidly growing mycobacteria (RGM) species. Infections in cystic fibrosis (CF) patients by this pathogen is a major health-concern worldwide^14,15^. The lack of optimal therapeutic treatments and the natural resistance of Mab to most available antibiotics^16–18^ has emphasized the need for the discovery of new strategies to overcome Mab infections.

A number of vaccine technologies have been applied in the attempt to develop new anti-tuberculosis live attenuated vaccines. There are 3 main strategies for the development of live attenuated mycobacterial vaccine including modifying BCG, attenuating Mtb or using nontuberculous mycobacteria (NTM) strains such as recombinant *Mycobacterium smegmatis* and *Mycobacterium indicus pranii* ^19–21^. Of note, genetically modified forms of *M. smegmatis* with deletions of the ESX-3 type VII secretion system have recently been developed and have demonstrated powerful induction of anti-mycobacterial immunity when injected into mice^22^, thereby highlighting the usefulness of the NTM strain as a live vaccine agent for tuberculosis.

Currently, several live vaccine candidates have been created by successive passaging in low-nutrition media, introducing genetic deletions, producing susceptibility to low or high temperatures, or engineering to require specific supplemental ingredients for growth. Theoretically, a live vaccine can proliferate and stay in the host for a sufficient duration to evoke a strong immune response but not long enough to express virulent phenotypes^23^. Temperature sensitive (TS) strains are widely used to create live human viral vaccines and have also been used to create some veterinary bacterial vaccines^24^. Recently, we introduced a temperature sensitive *Mycobacterium* spp., *Mycobacterium paragordonae* (Mpg), which can grow at permissive temperatures but fails to grow above 37 °C^25^. In this work, we tested the effectiveness of a naturally temperature sensitive mycobacterial strain, Mpg JCM 18565^T^, as a candidate for live vaccine for infections with Mab or Mtb.

## Results

### Attenuated infection of the temperature sensitive *M. paragordonae* (Mpg) in murine macrophages and an *in vivo* mouse model

**Figure 1:**
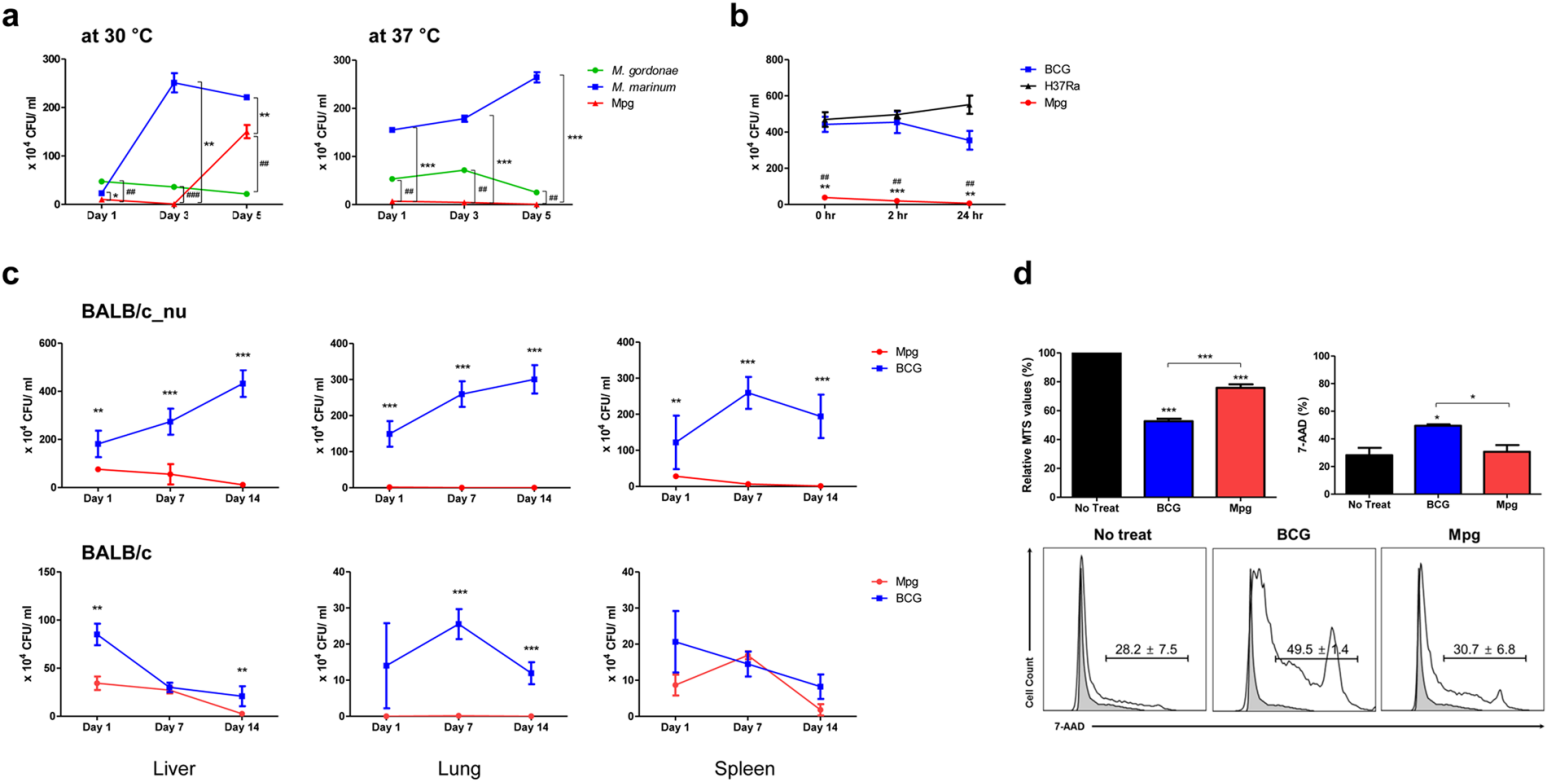
*M. paragordonae* (Mpg) led to the attenuated infection into murine macrophage and in an *in vivo* mice system. (**a**) Survival test of *M. gordonae*, *M. marinum* and Mpg strains (10 M.O.I. infection) at 30 °C (left) and 37 °C (right) in the murine macrophage J774.1. (**b**) Survival test of *M. bovis* BCG (BCG), Mpg and *M. tuberculosis* H37Ra (H37Ra) (10 M.O.I. infection) in the murine macrophage J774.1 at 37 °C in early time point (0, 2, and 24 hours). (**c**) Growth of BCG and Mpg in the organs (lungs, liver and spleen) after intravenous inoculation into BALB/c_nu (nude, up panels) and BALB/c (down panels) mice (n = 3–4 per group) (*P < 0.05, **P < 0.01, ***P < 0.001; Student’s *t*-test). (**d**) The viability of BMDCs was measured by MTS and 7-AAD staining assay after infection with 10 M.O.I. of BCG or Mpg overnight. Representative histograms of 7-AAD are presented (**P < 0.05, ***P < 0.001; Student’s *t*-test). All data are mean ± standard deviation of CFU or intensity values in each group.

To check the temperature sensitivity of Mpg during the infection of macrophages, we compared the colony forming units (CFUs) of Mpg in the murine macrophage cell line J774A.1 at different temperature (30 °C and 37 °C) with those of *M. gordonae*, which is the phylogenetically closest species to Mpg, and *M. marinum*, which is also known to have an optimal temperature of 30 °C as seen in Mpg^25^. Mpg could grow in the macrophages at 30 °C, but not at 37 °C. However, the other 2 mycobacteria could grow in the macrophages at both temperatures (Fig. 1a). Mpg showed significantly lower numbers of CFUs at all 3 time-points (1, 3, and 5 days) after infection at 37 °C compared to the other 2 mycobacteria. Nevertheless, Mpg showed a higher number of CFUs than *M. gordonae* 5 days after infection at 30 °C (Fig. 1a). This trend was also seen with BCG and Mtb (H37Ra), which showed significantly higher numbers of CFUs than did Mpg at all points after infection (Fig. 1b), thereby indicating a survival defect of Mpg in host infections under physiological conditions and providing a basis for minimizing the safety concern in its application in live vaccination.

To address the issue regarding low CFUs found in Mpg infected antigen presenting cells (APCs), transmission electron microscopic (TEM) images of Mpg infected J774A.1 cells were analyzed. The TEM image showed that Mpg was efficiently phagocytosed to J774A.1 cells (Supplementary Fig. S1). It suggests that Mpg has higher phagocytic capacity and low CFUs found in Mpg infected APCs may be due to its temperature sensitive trait, not due to its low phagocytic capacity.

To evaluate the safety of live Mpg in *in vivo* vaccination, we compared the bacterial burdens (CFUs) between Mpg and BCG in the different organs (liver, lungs and spleen) and at different time-points (1, 7, 14 and 28 days) after administering them into BALB/c and BALB/c_nu (nude) mice (1 × 10^6^ CFU, intravenous route) (Supplementary Fig. S2). In nude mice, the CFUs of Mpg in all the organs were significantly lower than those of BCG at each time point after infection. A similar trend was also found in the BALB/c mice, although some organ-specific differences were found (Fig. 1c). Even after 4 weeks of IV injection, the CFUs of Mpg were significantly lower than those of BCG in all the organs (Supplementary Fig. S3). It suggests usefulness of Mpg as an attenuated live vaccine.

It has been reported that during infections with mycobacteria, resistance against cell death of the APCs, particularly DCs can increase the efficacy of the vaccine by extending the duration of antigen presentation to the T cells^22^. To address this issue, 24 hours after the bone marrow-derived dendritic cells (BMDCs) from BALB/c mice were infected with BCG or Mpg at 37 °C, the cytotoxicity levels of the infected BMDCs were compared by MTS (cell supernatants) and 7-amino-actinomycin D (7-AAD) staining assays (cell pellets) capable of distinguishing between live and dead cells^26,27^. Both methods showed that the rates of death in the BMDCs were reduced after infection with Mpg compared to infection with BCG (Fig. 1d), suggesting that Mpg could be superior to BCG in extending the life-span of the infected DC, thereby facilitating antigen presentation.

### Enhancement of BMDC maturation by Mpg

**Figure 2:**
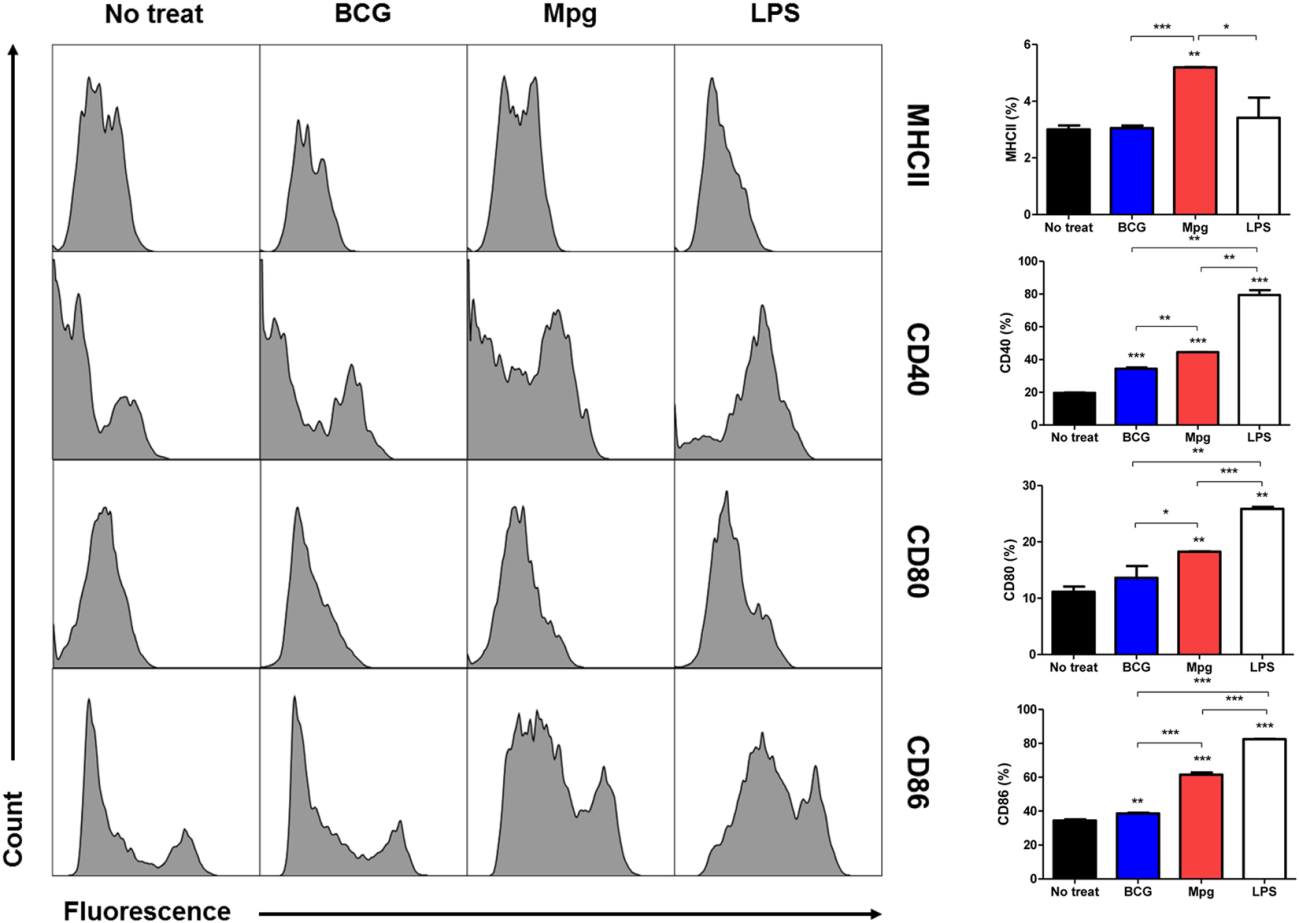
*M. paragordonae* (Mpg) induced enhanced BMDC maturation. BMDCs were infected with BCG, Mpg (10 M.O.I.) or LPS (1 μg/ml) overnight, stained with antibodies against DC maturation surface markers (CD40, CD80, CD86 and MHC II), and then analyzed by flow cytometer. Representative histograms of CD11c-gated cells are presented, and the mean fluorescent intensity (MFI) of each BMDC maturation surface marker are shown in the graph (*P < 0.05, **P < 0.01, ***P < 0.001; Student’s *t*-test).

To investigate the maturation of BMDCs after infection with Mpg, the immature BMDCs were infected with 10 M.O.I. of BCG or Mpg for 24 hours at 37 °C. As a positive control, the BMDCs were incubated with LPS (1 μg/ml) for 24 hours. The surface expression of maturation markers (MHC II, CD40, CD80 and CD86) on the BMDCs was compared between BCG- and Mpg-infected cells using FACS analysis (Fig. 2). All the DC maturation markers in the Mpg-infected BMDCs were increased compared to those in the uninfected (negative control) and BCG-infected BMDCs. In the case of MHC II, CD80 and CD86 surface markers, the BCG-infected BMDCs exhibited expression levels almost similar to the negative control, strongly supporting the previous report that BCG down-regulated MHC II and CD80 expressions in BMDCs^28^. This suggests that Mpg could induce the maturation of BMDCs compared to BCG.

**Figure 3:**
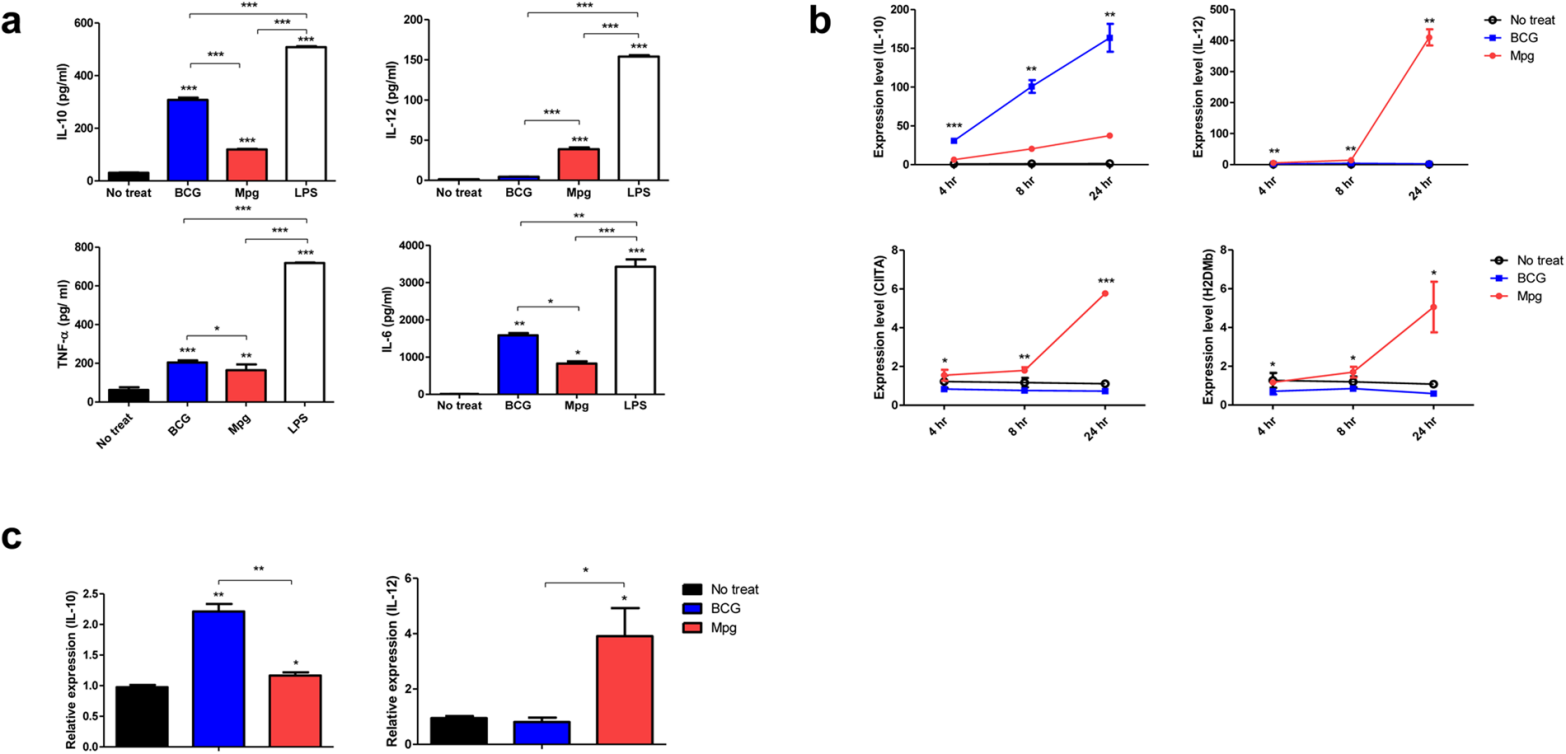
*M*. *paragordonae* (Mpg) induced BMDC or THP-1 -mediated immune responses. (**a**) Supernatants of the infected BMDCs were collected, and IFN-γ, TNF-α, IL-2, and IL-10 cytokine levels were analyzed by ELISA (*P < 0.05, **P < 0.01, ***P < 0.001; Student’s *t*-test). (**b**) qRT-PCR was used to measure the expression levels of IL-10, IL-12, CIITA and H2DMb mRNA in the BMDCs infected with BCG or Mpg. The expression levels represent relative fold changes based on β-actin. (**c**) qRT-PCR was used to measure the expression levels of IL-10 and IL-12 mRNA in the THP-1 cells infected with BCG or Mpg. The expression levels represent relative fold changes based on 18S rRNA. (*P < 0.05, **P < 0.01, ***P < 0.001; Student’s *t*-test).

To compare the cytokine profiles of BMDCs after infection with BCG or Mpg, the cell culture supernatants were subject to ELISA (IL-10, IL-12, TNF-α or IL-6 cytokines). Notably, the Mpg-infected BMDCs produced significantly higher levels of IL-12 cytokines than the BCG- infected BMDCs. However, the Mpg-infected BMDCs secreted significantly lower levels of the other 3 cytokines TNF-α, IL-6, and IL-10 than the BCG-infected BMDCs (Fig. 3a). Given that the production of cytokines such as IL-10 and IL-12 during the process of BMDC maturation process can induce a Th1- or Th2-skewed immune response in the context of local inflammation^29–31^, our data suggest that Mpg could elicit a Th1-type immune response compared to BCG. These results were further supported by qRT-PCR analysis. The mRNA levels of IL-10 or IL-12 and two molecules related to MHC II expression, CIITA (MHC II transactivator) and H2DMb (MHC II-associated protein), were compared between the BCG- and Mpg-infected BMDCs. Consistent with the ELISA data, IL-10 expression was significantly enhanced in the BCG-infected BMDCs, compared to the Mpg-infected BMDC, irrespective of the time after infection (about five-fold increase compared with Mpg-infected BMDCs at each time-point). In addition, the Mpg-infected BMDCs showed significantly higher expression levels of IL-12 than the BCG-infected BMDCs at 24 hours after infection (about one hundred-fold increase compared with the BCG-infected BMDCs) (Fig. 3b). A similar trend as shown in murine cell lines was also found in IL-10 and IL-12 cytokine profiles of Mpg infected human monocyte cell line, THP-1 (Fig. 3c). Additionally, the Mpg-infected BMDCs showed higher levels of the two MHC II-related targets, CIITA and H2DMb, than the BCG-infected BMDCs (about five-fold increase compared with the BCG-infected BMDCs 24 hours after infection) (Fig. 3b). In the case of CIITA, the CIITA mRNA level was increased in 24 hours, but reduced in 48 hours after infection with Mpg (Supplementary Fig. S4), which is consistent with the previous finding that the CIITA mRNA level of Mtb infected cells was increased in 24 hours and decreased in 48 hours^32^.

### Enhanced BMDC migration by Mpg

**Figure 4:**
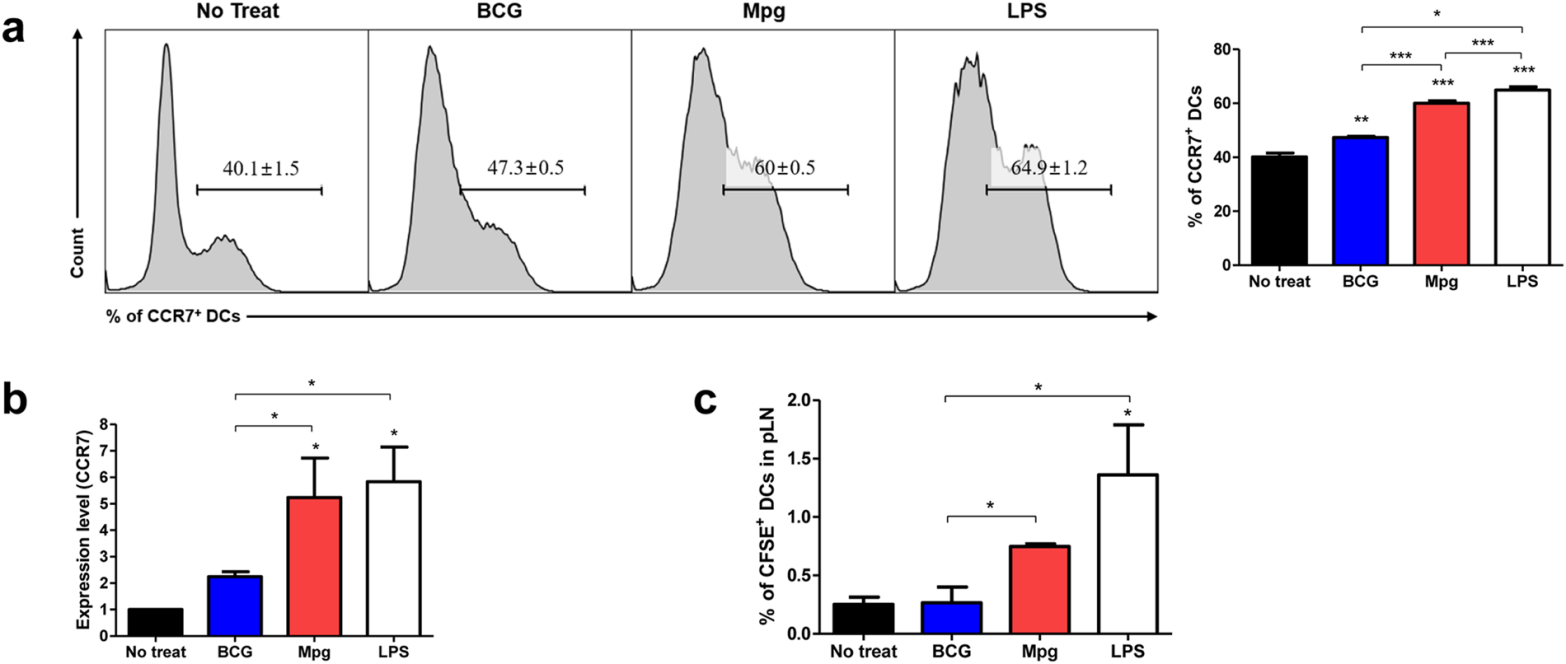
*M. paragordonae* (Mpg) induced enhanced BMDC migration. Expression levels of CCR7 protein and mRNA were measured by (**a**) flow cytometry and (**b**) qRT-PCR, respectively. To show the rate of CCR7^+^ BMDCs, representative histograms of CD11c-gated cells are presented and the expression levels denote the relative fold changes based on β-actin (*P < 0.05, **P < 0.01, ***P < 0.001; Student’s *t*-test). (**c**) *In vivo* migration of the infected BMDC with BCG or Mpg was measured by comparisons of CFSE-labeled BMDCs in popliteal lymph nodes (pLN) 2 days after injection of the BCG- or Mpg-infected BMDCs into mice footpad. The CFSE-labeled BMDCs among the CD11c ^+^ cells in popliteal lymph node were measured by flow cytometry. The mean percentile of the CFSE-labeled BMDCs in the popliteal lymph nodes from separated experiments are presented (*P < 0.05; Student’s *t*-test).

Since the migration of BMDCs to regional lymph nodes requires the expression of CCR7 as the receptor for lymphoid chemokines CCL19 and CCL2^33^, we compared the expression of CCR7 between BMDCs infected with either BCG or Mpg. To evaluate the migrational abilities of the infected BMDCs with BCG or Mpg, we compared the level of CCR7 expression on infected cell surfaces using FACS analysis. We found that the surface expression of CCR7 on the Mpg-infected BMDCs were significantly higher than in the BCG-infected BMDCs (Fig. 4a), and this result was also consistent with the qRT-PCR quantification of the corresponding mRNA level (Fig. 4b). To further evaluate the migrational capacity of BMDCs in response to Mpg, we compared the entry of BMDCs into lymphatic vessels between the BMDCs infected with either BCG or Mpg. We investigated the migration of the CFSE-labeled BMDCs stimulated with 10 M.O.I. BCG, Mpg or LPS (1 μg/ ml) after they were injected into the footpads of mice. Our data showed that the Mpg-infected BMDCs were more than twice as likely to migrate to the pLNs than the BCG-infected BMDCs (*P* < 0.05) (Fig. 4c). Taken together, our data suggest that Mpg could also induce enhanced BMDC migration compared to BCG.

### BMDC infected with Mpg elicited enhanced mycobacteria specific CD4 or CD8 T cell response

**Figure 5:**
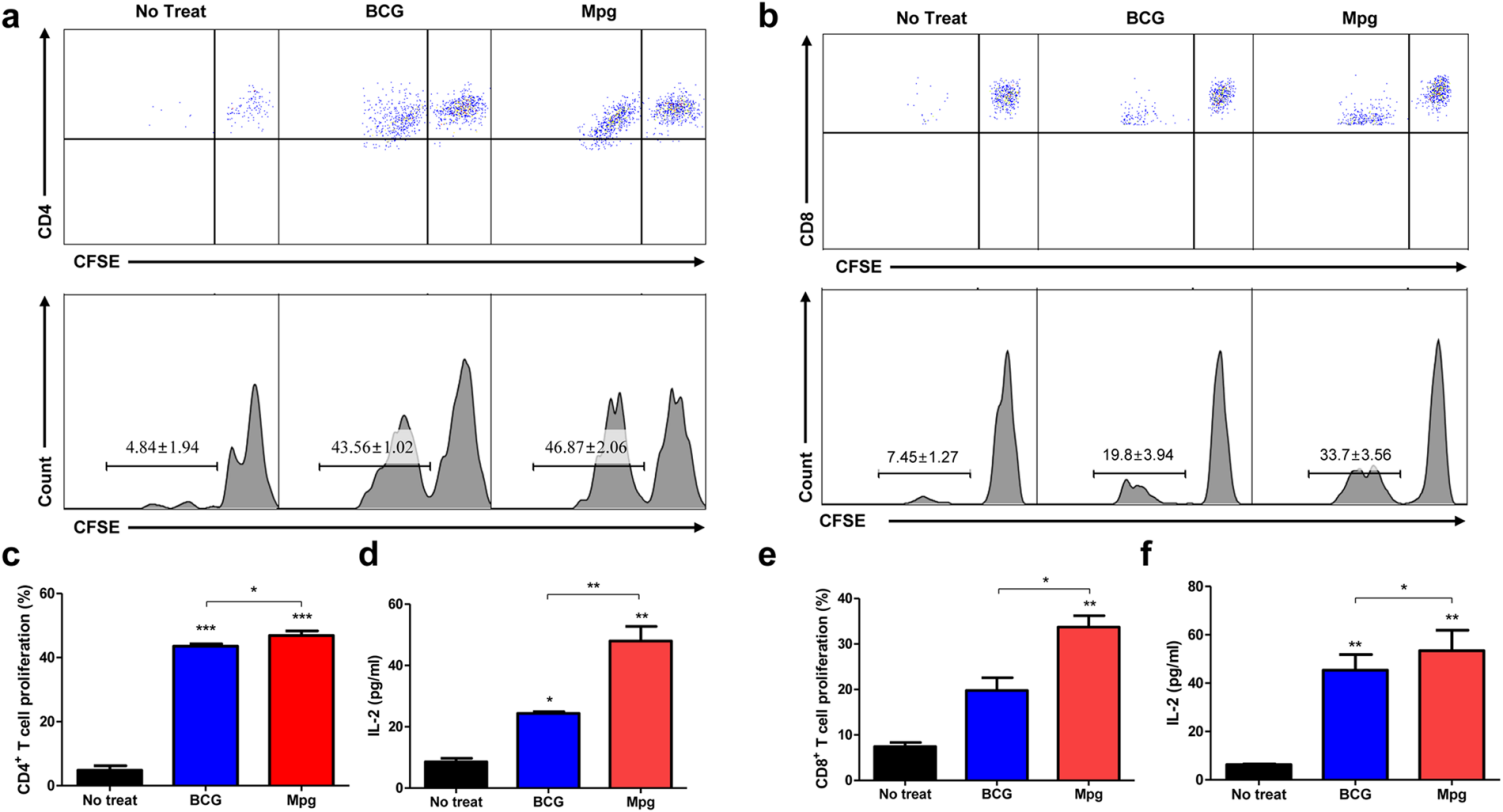
Enhanced T cell proliferation induced by *M. paragordonae* (Mpg)-infected BMDCs. Proliferation of (**a** and **c**) CD4 and (**b** and **e**) CD8 T cells induced by BCG- or Mpg-infected BMDCs was examined by CFSE dilution method and detected by flow cytometry. The data represent the mean ± standard deviation of CFSE intensities in each group (*P < 0.05, **P < 0.01, ***P < 0.001; Student’s *t*-test). (**d** and **f**) IL-2 cytokine amounts in the supernatants of the CD4 and CD8 T cells used for the proliferation assay were measured by ELISA (*P < 0.05, **P < 0.01, ***P < 0.001; Student’s *t*-test).

To test whether Mpg could have an effect on inducing adaptive immune responses, especially to promote T cell proliferation against mycobacteria, we conducted T cell proliferation assay by the CFSE dilution method^34^. The results showed that the BMDCs infected with Mpg could induce significantly increased proliferation levels in the both CD4 and CD8 T cells compared to the BMDCs infected with BCG (Fig. 5a–c and e). In fact, the increases in proliferation in response to BMDCs infected with Mpg or BCG were more pronounced for the CD8 T cells (Fig. 5c and e). Additionally, the amounts of secreted IL-2 cytokine from the stimulated CD4 and CD8 T cells were compared. As shown by FACS analysis, both CD4 and CD8 T cells stimulated with the Mpg-infected BMDCs secreted significantly higher levels of IL-2 than the BCG-infected BMDCs (Fig. 5d and f). This suggests that the BMDCs infected with Mpg could exert more efficient mycobacteria-specific T cell immune responses than the BMDCs infected with BCG.

### Mpg elicits an enhanced cell-mediated immune response against Mab or Mtb infections in vaccinated mice

The above findings demonstrated that Mpg has the potential to function as a live vaccine against mycobacterial infection. Therefore, to address the issue, we sought to check the protective effect of Mpg against infections with two different mycobacteria, Mab and Mtb. The mice were divided into 4 groups and immunized twice by prime-boosting (4 weeks after priming vaccination) protocol (Supplementary Fig. S2).

**Figure 6:**
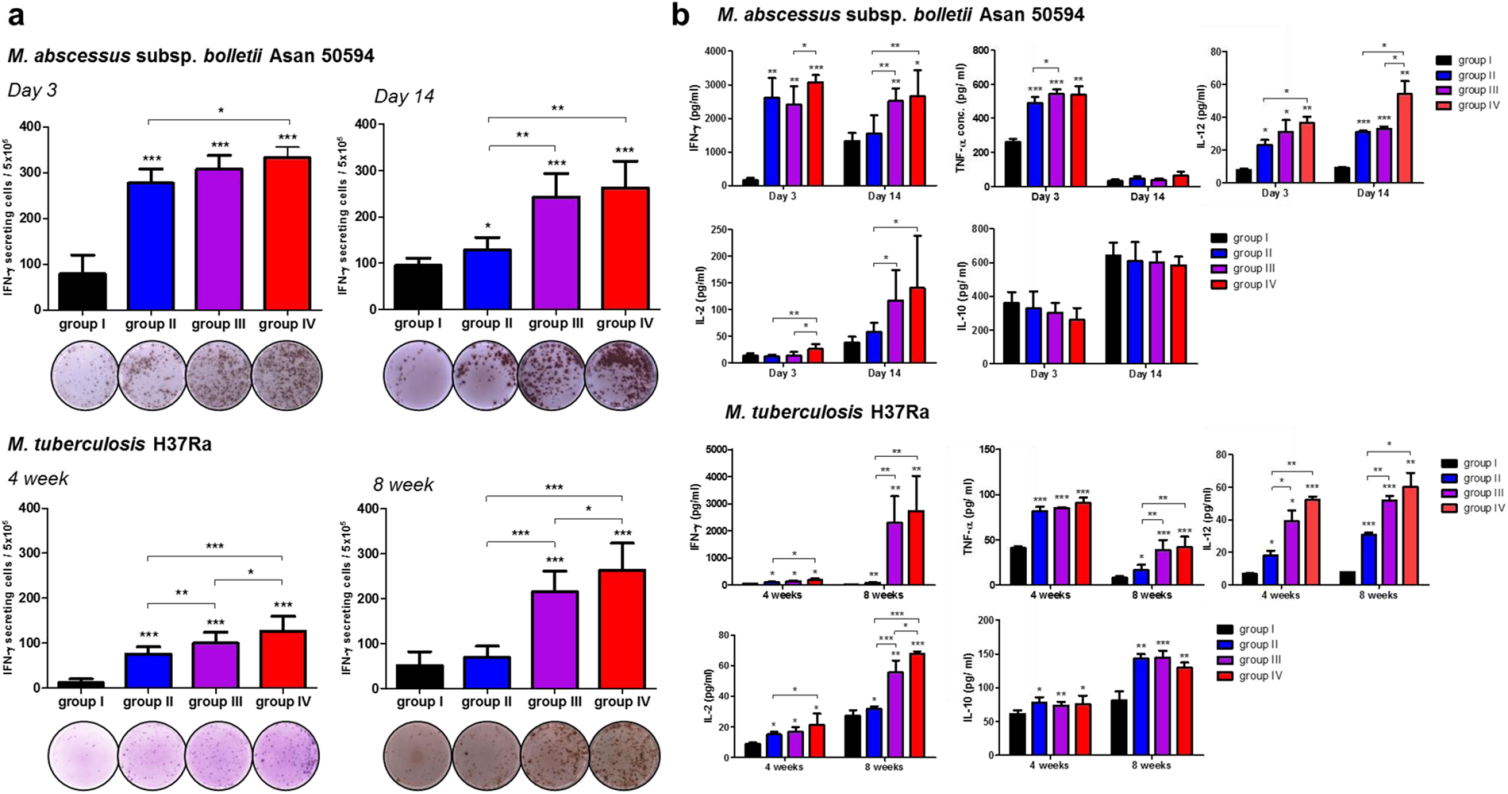
*M*. *paragordonae* (Mpg) elicits an enhanced cell mediated immune response against *M. abscessus* or tuberculosis infections in vaccinated mice. (**a**) Splenocytes from vaccinated (with BCG or Mpg) and challenged (with Mab or Mtb) mice (n = 3–5 per group) were stimulated with whole cell lysates of Mab and Mtb. The IFN-γ secretion levels were detected by ELISPOT analysis (*P < 0.05, **P < 0.01, ***P < 0.001; Student’s *t*-test). (**b**) IFN-γ, TNF-α, IL-2, IL-10 and IL-12 cytokine levels were detected from the culture supernatants of the *in vitro* stimulated splenocytes with whole cell lysates of Mab and Mtb by ELISA (*P < 0.05, **P < 0.01, ***P < 0.001; Student’s *t*-test). Each vaccinated group is annotated as follows: group I [PBS + PBS], group II [BCG + BCG], group III [BCG + Mpg], and group IV [Mpg + Mpg].

To test whether Mpg could improve the cell-mediated immune (CMI) response after vaccination, the splenocytes of mice from each vaccinated group were subjected to IFN-γ ELISPOT analysis. In the vaccinated mice challenged with Mab, the two groups with Mpg in the vaccine module, groups III [BCG + Mpg] and IV [Mpg + Mpg], showed a significantly more robust IFN-γ secretion and yielded significantly higher numbers of IFN-γ spot forming cells (SFCs) compared to groups I [PBS + PBS] or II [BCG + BCG] at 3 or 14 days after infection. This trend was also seen in the case of Mtb challenge. Of note, group IV showed a significantly more robust IFN-γ secretion and yielded significantly higher numbers of IFN-γ SPCs than group III at 4 or 8 weeks after infection (Fig. 6a). Therefore, the increase in the use of Mpg to the vaccine module (0 vs. 1 vs. 2) was correlated with the potentiation of the CMI response in the Mtb-challenged mice, thereby strongly suggesting a significant role of Mpg in eliciting CMI response against mycobacteria. Additionally, secreted cytokine levels of IFN-γ, TNF-α, IL-2, IL-10 and IL-12 were detected by ELISA in the supernatants of the cultured splenocytes (stimulated with whole cell lysates of Mab and Mtb). The levels of secreted IFN-γ were correlated with the IFN-γ ELISPOT results. Of note, in the Mtb-challenged mice 8 weeks after infection, the difference between the vaccinated groups with or without Mpg as the vaccine module was the most pronounced. In the Mtb-challenged mice, the two vaccinated groups with Mpg as the vaccine module, groups III and IV, secreted significantly higher levels of TNF-α compared to the other groups without Mpg as the vaccine module, groups I and II at 8 weeks after infection. In the Mab- or Mtb-challenged mice, group IV always produced a significantly higher level of secreted IL-2 compared to other groups without Mpg, groups I and II. Also, in the case of IL-12 expression, group IV of Mtb- and Mab-challenged mice produced a significantly higher level of IL-12 compared to groups II and/or III. In the Mab- or Mtb-challenged mice almost similar levels of the anti-inflammatory cytokine IL-10 was found in groups II, III and IV (Fig. 6b). These data showed that Mpg vaccination enhanced the secretion of Th1 immune response-related cytokines (IFN-γ, TNF-α, IL-2 and IL-12) compared to BCG vaccination.

### Mpg elicits a mycobacterial-specific Th1-biased humoral response in immunized mice

**Figure 7:**
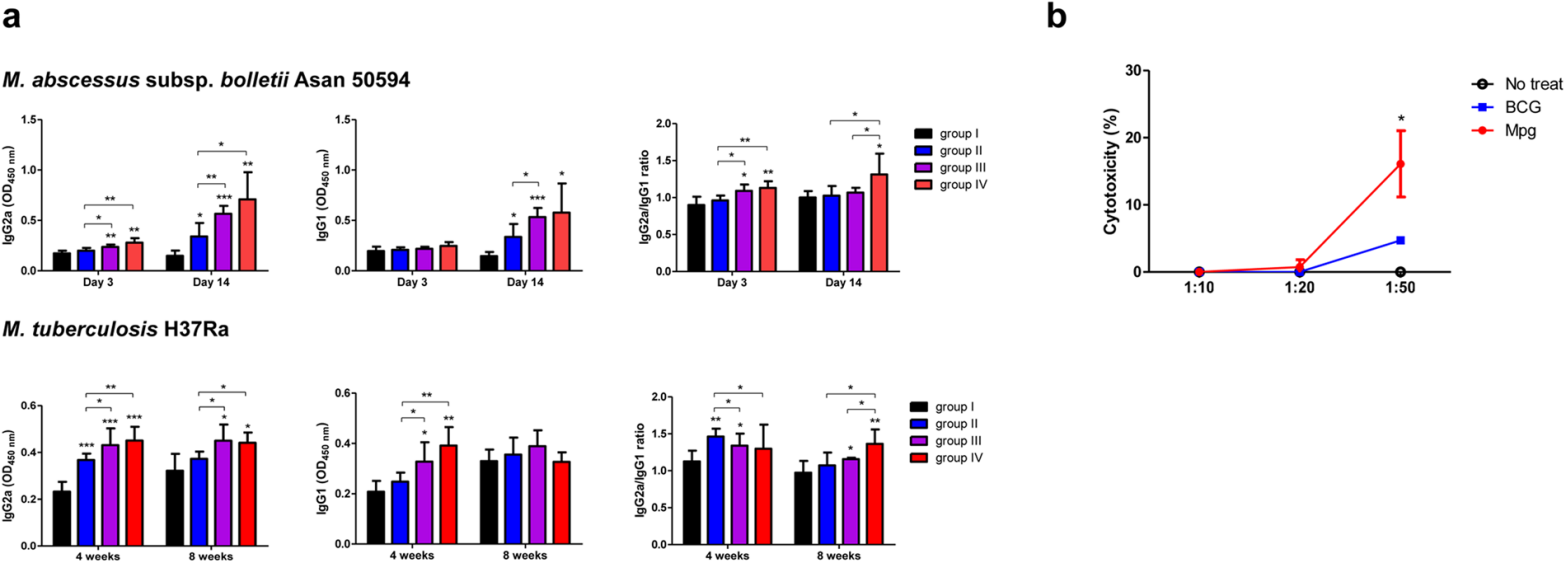
*M. paragordonae* (Mpg) elicits an enhanced mycobacterial-specific Th1-biased humoral response and cytotoxic T lymphocyte (CTL) response in immunized mice. (**a**) Mab- and Mtb-specific immunoglobulin subtypes (IgG2a and IgG1) were detected by ELISA from serum samples of vaccinated and challenged mice. Optical densities at 450 nm of IgG2a and IgG1 isotypes were detected and compared among all the vaccinated groups at each time point. The ratio of IgG2a/IgG1 was calculated by dividing the OD values for IgG2a by OD values for IgG1 (*P < 0.05, **P < 0.01, ***P < 0.001; Student’s *t*-test). (**b**) Splenocytes from BCG- or Mpg-immunized mice were stimulated with Ag85B protein for 6 days and used as effector cells. P815 cells (H-2^d^) were transfected with pcDNA 3.3-Ag85B:ESAT-6 expression vector and used as target cells. CTL assay was conducted by co-culturing the target and effector cells at various ratio (E/T ratios of 10:1, 20:1, and 50:1). Supernatants of co-cultured cells were used for lactate dehydrogenase (LDH) cytotoxicity assay (*P < 0.05; Student’s *t*-test). Each vaccinated group is annotated as follows: group I [PBS + PBS], group II [BCG + BCG], group III [BCG + Mpg], and group IV [Mpg + Mpg].

To examine whether Mpg vaccination also elicits a Th1-biased humoral immune response after the challenge with Mab or Mtb, we analyzed the levels of Mab- or Mtb-specific IgG2a and IgG1 isotypes in the serum samples from the vaccinated mice. IgG2a and IgG1 are known representatives of Th1 and Th2 immune response markers, respectively^35–37^. As shown in Fig. 6a, in the Mab- or Mtb-challenged mice, the two vaccinated group with Mpg as the vaccine module groups III and IV secreted significantly higher levels of IgG2a compared to the other groups without Mpg, groups I and II. The two vaccinated groups with Mpg as the vaccine module groups III and IV also secreted significantly higher levels of IgG1 than groups I and II in the Mab-challenged mice 14 days after infection or in the Mtb-challenged mice 4 weeks after infection. Of note, the highest IgG2a/IgG1 ratio indicative of a Th1-biased humoral immune response^35–37^ was found in group IV in the Mab-challenged mice 14 days after infection or in the Mtb-challenged mice 8 weeks after infection (Fig. 7a). These results suggested that Mpg could elicit an enhanced Th1-biased humoral immune response in the vaccinated mice.

### Mpg elicits a mycobacterial-specific cytotoxic T lymphocyte response in immunized mice

Additionally, to test whether Mpg elicits an enhanced mycobacteria-specific cytotoxic T lymphocyte (CTL) response in the vaccinated mice, we analyzed CTL activities of splenocytes from mice vaccinated with BCG or Mpg via lactate dehydrogenase (LDH) cytotoxicity assay. The immunization procedure used a prime-boost protocol (boost vaccination 2 weeks after prime vaccination: BCG + BCG or Mpg + Mpg) as described in Supplementary Fig. 2c. The splenocytes from mice immunized with BCG or Mpg were stimulated *in vitro* with Ag85B protein (5 μg/ml) for 6 days and used as effector cells. P815 cells (H-2^d^) transfected with the plasmid harboring Ag85B-ESAT-6 fusion genes (pcDNA3.3-Ag85B:ESAT-6) served as target cells^38^. As shown in Fig. 7b, at the E/T ratio of 50:1, the CTLs from the Mpg-immunized mouse splenocytes elicited significantly higher levels of Ag85B specific target cell lysis compared to the BCG-immunized mouse splenocytes (*P* < 0.05). Our data indicated that Mpg can elicit an enhanced mycobacterial-specific CTL response in the immunized mice.

### Mpg elicited an enhanced protective vaccine efficacy against Mab or Mtb infection

**Figure 8:**
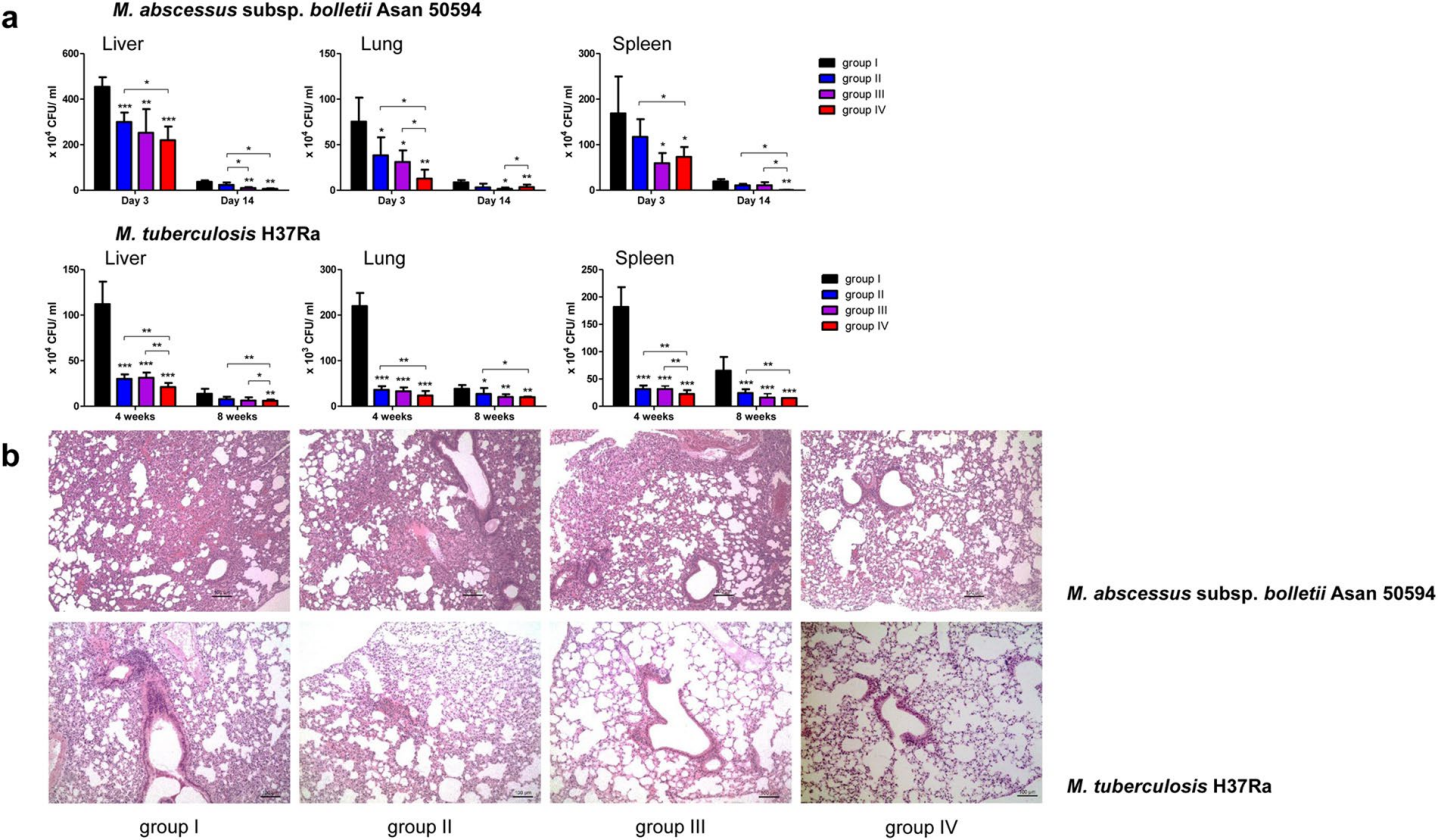
*M. paragordonae* (Mpg) vaccine elicited an enhanced protective efficacy against *M. abscessus* or *M. tuberculosis* infections in a mouse model. (**a**) CFU enumeration from organs (liver, lung and spleen) of BALB/c mice (n = 4–6 per group) vaccinated subcutaneously with PBS, BCG (1 × 10^6^ CFU) or Mpg (1 × 10^6^ CFU) (vaccination groups are detailed in Supplementary Fig. 2) and challenged 4 weeks after final vaccination with Mab or Mtb (1 × 10^6^ CFU, intravenously) (*P <b 0.05, **P < 0.01, ***P < 0.001; Student’s *t*-test). (**b**) Representative histopathologic images (H&E stained) are shown for the sectioned lung tissues of BALB/c mice vaccinated with PBS, BCG or Mpg (vaccination groups are detailed in Supplementary Fig. 2) and challenged with Mab (at Day 14) or Mtb (at Week 8). Scale bars correspond to 100 μm. Each vaccinated group is annotated as follows: group I [PBS + PBS], group II [BCG + BCG], group III [BCG + Mpg], and group IV [Mpg + Mpg].

To examine the protective immune response against Mab and Mtb after vaccination with Mpg, organs (liver, lung and spleen) from mice challenged with Mab or Mtb strains after vaccination were homogenized at each time point (3 and 14 days after challenge with Mab; 4 and 8 weeks after challenge with Mtb), and their CFUs were calculated. Mpg vaccination resulted in significant reductions in bacterial burdens (both Mab and Mtb) in all the organs that compared BCG vaccination at all time-points. Notably, in the mice challenged with Mab or Mtb, group IV always showed significantly lower levels of bacterial burden in all organs or at both time-points compared to groups I or II, except for the lungs of the mice challenged with Mab 14 days after infection (Fig. 8a). Histopathological evaluation further showed superior protective immunity after Mpg vaccination compared to BCG vaccination. More resolved inflammation was found in the lung tissues form the vaccinated mice in the two groups vaccinated with Mpg, groups III and IV, compared to groups I or II (Fig. 8b). Notably, the recovery of inflammation was most pronounced in group IV that used Mpg both for priming and boosting vaccination (Fig. 8b). Taken together, our data suggest that Mpg can elicit an enhanced vaccine efficacy in immunized mice.

## Discussion

In the current study, we have demonstrated that compared to BCG, a temperature sensitive Mpg plays a pivotal role as an attenuated live vaccine in protecting against mycobacterial infections with not only Mtb, but also Mab. Our finding that Mpg fails to infect macrophages at 37 °C and elicits a milder infection in mice compared to BCG (Fig. 1c) provides a novel insight into why its isolation frequency from clinical specimens is rare. Indeed, Mpg was serendipitously discovered from the sputum sample of a Korean patient who was infected with Mab by a culture at room temperature and not at 37 °C by our groups^25^. To the best of our knowledge, other Mpg strains except for Mpg JCM 18565^T^ have not been found to date, suggesting that Mpg may be a genuinely rare NTM that is generally present in the environment or in colder regions or tissues of body such as mucous membranes and lacks the capacity to infect deeper tissues of the human body. The bacteriological, epidemiological or clinical traits of Mpg led us to hypothesize that it could function as the first temperature sensitive strain for live vaccination to prevent mycobacterial diseases. The naturally present TS strain of Mpg may have a very low chance of reversion into a temperature-insensitive phenotype, which is a frequent occurrence with artificially modified TS mutants, and thus Mpg could be much safer as a live vaccine than the artificially modified strains.

**Figure 9:**
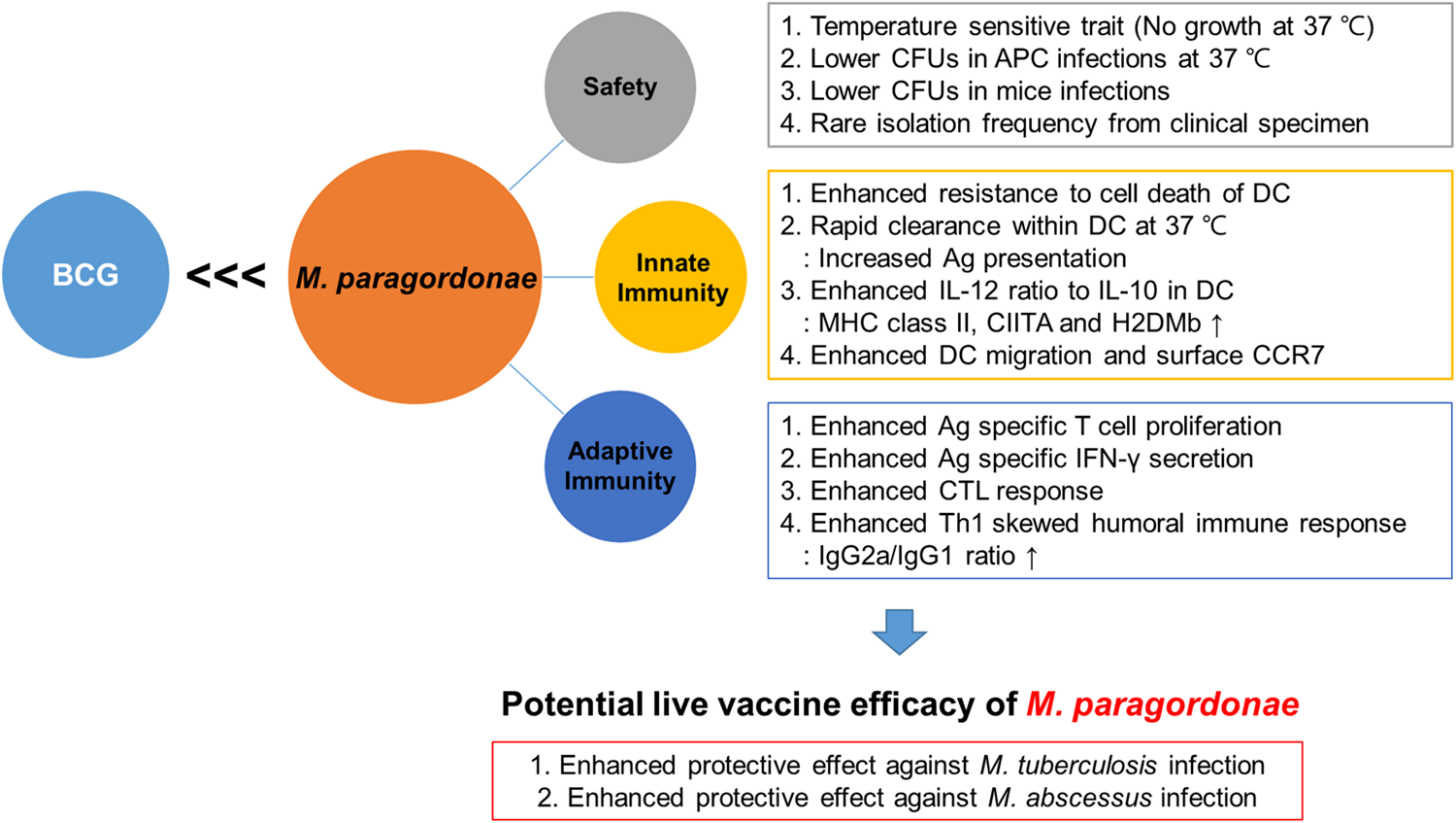
Schematic representation showing enhanced vaccine efficacy of *M. paragordonae* (Mpg). Compared to BCG, Mpg plays a pivotal role as an attenuated live vaccine in protecting against infections with Mtb and Mab via enhanced innate and adaptive immune responses and is safer for mice intravenous infections as well as in infections of macrophages.

Our findings supporting the superiority of Mpg to BCG in eliciting DC maturation and contributing into vaccine efficacy against mycobacterial diseases are noteworthy (Fig. 9). First, immature BMDCs infected with Mpg are more resistant to cell death, which could facilitate the presentation of the mycobacterial antigen to the T cells. This is consistent with a previous report, which demonstrated that the extended survival of DCs could enhance the efficacy of the vaccine^39^. Second, the reduced survival of Mpg within DCs at the physiological temperature of 37 °C could provide increased mycobacterial peptide production from the digested Mpg into the T cells, thereby leading to enhanced mycobacteria specific T cell proliferation and activation. Indeed, our data also showed that the BMDCs infected with Mpg could induce significantly higher levels of proliferation of the Mtb antigen-specific CD4 or CD8 T cells compared to the BMDCs infected with BCG (Fig. 5), thereby supporting the above notion. Furthermore, there are several reports supporting a possible role of the deleted mutant of BCG or Mtb interfering with its persistent survival in the APCs as a candidate for live attenuated vaccines^40–45^. Third, our data also showed that the innate immune response elicited by a more attenuated Mpg strain generates a cytokine milieu that favors the priming of Th1 cell responses, indicated by the enhanced ratio of the Th1 stimulator IL-12 to the immune regulatory IL-10 (Fig. 3). Since IL-10 can be acted as a negative regulator of induced IL-12 production^46^, both IL-10 and IL-12 are together induced in LPS treated BMDCs, in a TLR4 dependent manner^47^. However, in Mpg infected BMDC, the relative higher level of IL-12 compared to IL-10 is induced (Fig. 3). This IL-12 inducing capacity of Mpg, in turn, can lead to an enhanced adaptive immunity, which may be directed against the Mpg-encoded antigens that are shared with Mab or Mtb. Indeed, our data using vaccinated mice also showed that Mpg elicits a Th1-skewed adaptive immune response as shown by an enhanced mycobacterial-specific IFN-γ secretion (Fig. 6), IgG2a/IgG1 ratio (Fig. 7a) and CTL response (Fig. 7b). In particular, it has also been reported that the infection of BCG or Mtb into DCs could lead to enhanced secretion of immune regulatory IL-10 via TLR2-dependent ERK activation, reciprocally suppressing IL-12 secretion^48^. It can reduce the adaptive immune response against mycobacterial antigens, which is the main weakness of BCG as a live vaccine against mycobacterial diseases^48,49^. The higher level of IL-12 to IL-10 ratio found in the BMDCs infected with Mpg compared to those infected with BCG could lead to an increased expression of genes involved in antigen presentation, such as MHC class II, CIITA or H2DMb as shown by our data (Fig. 3). Fourth, we showed that Mpg could also induce an enhanced BMDC migration compared to BCG by measuring the surface expression of CCR7 in the Mpg-infected BMDCs (Fig. 4a) and by quantifying the DCs that had migrated from the footpad into the pLN (Fig. 4b). This can definitely increase the chances of antigen presenting DCs to encounter antigen-specific CD4 or CD8 T cells in the draining lymph node.

Notably, our finding that the addition of Mpg in our prime-booster immunized protocols positively correlated with improvement of the CMI response (Fig. 6) and protective immune response against Mab or Mtb reveals the superiority of Mpg over BCG in the efficacy of the vaccine against mycobacterial diseases (Fig. 8). Particularly, our result showing improved CMI or protective immune response in group III [BCG + Mpg] compared to group II [BCG + BCG] (Fig. 6) suggests a potential for Mpg to be developed not only as a priming vaccine in BCG non vaccinated (naïve) humans but also as a booster vaccine in BCG-vaccinated humans. Our IFN-γ ELISPOT data showed that the CMI enhancing effect of Mpg is more pronounced in the boosting stage than in the priming stage in Mab or Mtb infections compared to that of BCG (Fig. 6a). In particular, the boosting effect of BCG was almost marginal, suggesting that there may be a disparity between Mpg and BCG in inducing memory T cell responses. This further supports the notion of the potential of Mpg as a booster vaccine against Mtb infections in BCG-vaccinated adults. The issue of how Mpg could exert a CMI boosting effect via modulation of memory T cells should be addressed in the future.

The need for vaccines capable of protecting against Mab infections has increased because of the growing number of cases around a world that are failing treatment due to the natural resistance of Mab to most of the available antibiotics^16–18^. However, very few studies regarding vaccine development against Mab have been reported to date. The ones that have been reported mainly focus on DNA vaccine target genes, such as phospholipase C^50^ or MgtC^51^, related to the virulence of Mab. Our data clearly demonstrated the utility of Mpg as a live attenuated vaccine against Mab and Mtb infections. Particularly, the strong inducing effect of Mpg in eliciting a CMI response against Mab infection in the vaccinated mice belonging to group III [BCG + Mpg] (Fig. 6) shown by our data suggests its potential role as a booster live vaccine in BCG-vaccinated adults, which is a major target population for vaccination. To the best of our knowledge, this is the first report showing the use of a live attenuated vaccine against Mab.

Usefulness of NTMs such as heat killed or live *M. vaccae* and *M. indicus pranii* as TB vaccine have been widely studies so far^52,53^. Both *M. vaccae* and *M. indicus pranii* showed enhanced CD8^+^ cytotoxic T lymphocyte (CTL) responses and the expression of IFN-γ against TB in mouse model^54,55^. Also, they contribute to reduce the TB burdens in mouse^55,56^. However, most of studies mainly focused on their immunotherapeutic capacity using heat killed strains^57,58^, but their potential as preventive vaccines using live attenuated strains are still controversial. In the current study, we clearly proved usefulness of Mpg as a live attenuated preventive vaccine against mycobacterial infections. We think that capacity to elicit an enhanced innate immune response in infected APCs and temperature sensitive trait of Mpg may provide a better potential for development as a live attenuated vaccine for mycobacterial infections, compared to other NTMs.

There are some noteworthy limitations in this study. First, the mice used for the evaluation of the efficacy of the vaccine against Mtb infections were infected with avirulent H37Ra strain instead of virulent strains such as H37Rv or clinical isolates. Second, an intravenous challenge model was used for vaccine evaluation instead of an aerosol challenge model, which is the main route of infection with Mab or Mtb. We have plans for evaluating the vaccine efficacy against Mtb with an aerosol challenge model using virulent Mtb strains in the future. We are also currently in the process of improving the vaccine efficacy of Mpg for its future implementation as a vaccine in human via its combination with other types of vaccine modules in prime-boost regimens or via using different immunization routes including the intranasal route.

In conclusion, our data suggest that a temperature sensitive Mpg may be a potentially powerful candidate vaccine strain for inducing enhanced protective immune responses against mycobacterial infections.

## Materials and Methods

### Mycobacterial strains and culture conditions

The mycobacterial strains used in this study are as follows: *Mycobacterium abscessus* subsp. *bolletii* Asan 50594 (Mab)^59,60^, *M. bovis* BCG Tokyo strain (BCG), *M. gordonae* ATCC 14470^T^, *M. marinum* JCM 17638^T^, *M. paragordonae* JCM 18565^T^ (Mpg)^25^ and *M. tuberculosis* (Mtb) H37Ra ATCC 25177. All the strains were cultured from low-passage frozen stocks (at −70 °C) to exponential phase and subcultured in 7H9 broth (supplemented with 10% ADC) or 7H10 agar plate (supplemented with 10% OADC) at 30 °C (in the case of Mpg) or 37 °C for each experiment. To obtain single bacterial cell suspensions, all the strains were suspended in PBS with 0.05% Tween 80 (PBS-T) and passed through a 27-gauge needle three to five times.

### Cell cultures and infection with mycobacterial strains

The murine macrophage cell line, J774A.1 (American Type Culture Collection, ATCC TIB-67) was maintained at 37 °C and 5% CO_2_ in Dulbecco’s modified Eagle’s medium (DMEM; Thermo Scientific, Rockford, IL, USA) supplemented with 10% (v/v) fetal bovine serum (FBS), 2 mM glutamine, and essential amino acids. The bone marrow-derived dendritic cells (BMDCs) were generated using the femurs and tibias from 6- to 10-week-old BALB/c mice as described previously^38,61^. The J774A.1 and immature BMDCs were infected with mycobacterial strains at a multiplicity of infection (M.O.I.) of 10 and incubated for 4 hours to allow the phagocytosis of the bacteria. The extracellular bacteria were subsequently removed by washing three times with PBS followed by the addition of fresh media. After further incubation, the cells were detached by PBS with 0.5% Triton-X-100 or scraping at each time point. The cell pellets were diluted in PBS and plated onto 7H10 agar plates (supplemented with OADC) to determine the colony forming units (CFUs). The cell culture supernatants were collected and stored in the deep freezer (at −70 °C) for detecting the cytokine levels.

### MTS and 7-AAD staining assays for the measurement of viability of BMDCs

Viability of the infected BMDCs was measured by MTS assay (CellTiter 96® Aqueous One Solution Cell Proliferation Assay; Promega, Madison, WI, USA). BMDCs were infected or treated with 10 M.O.I. of BCG, Mpg or LPS (1 μg/ ml) overnight. After infection, the BMDCs were washed 3 times with PBS and equal numbers of BMDCs were suspended in 100 μl of IMDM. Then, 20 μl of MTS solution was added directly to each well and incubated for 4 hours at 37 °C. To analyze cell viability by MTS reduction colorimetrically, absorbance was measured at 490 nm (TECAN Infinite M200 PRO).

To detect the dead BMDCs, we used the 7-AAD staining assay. The BMDCs were infected as before. The infected BMDCs were washed three times with cold PBS and resuspended in 100 μl of cold FACS buffer (1% FBS and 5 mM EDTA in PBS). The suspended BMDCs were stained with 7-AAD for 5 min and immediately analyzed by flow cytometry (BD LSRFortessa).

### Flow cytometry assay for BMDC maturation

The infected BMDCs were washed three times with ice-cold PBS and blocked on ice for 30 min with super block solution containing 10% mouse sera (Sigma Aldrich), 10% rat sera (Sigma Aldrich), 10% goat sera (Gibco Invitrogen), and 2.4G2 monoclonal antibody (10 μg/ml, Invitrogen). The blocked BMDCs were stained with APC-Cy7-conjugated anti-CD11c (clone N418; BD Pharmingen), FITC-conjugated anti-I-A^b^ (clone AF6-120.1; BD Pharmingen), PE conjugated anti-CD40 (clone 3/23; BD Pharmingen), APC-conjugated anti-CD80 (clone 16-10A1; eBioscience) and PE/Cy7-conjugated anti-CD86 (clone GL-1; BioLegend) for 30 min on ice and washed three times with cold PBS. The stained BMDCs were resuspended in FACS buffer (PBS with 1% bovine serum albumin and 1 mM EDTA). The BMDCs were analyzed by flow cytometry (BD LSRFortessa). The data were analyzed with FlowJo software version 10.1 (FlowJo, Ashland, OR, USA).

### DC migration assay

To examine the migration of the infected BMDCs from the footpad to popliteal lymph node (pLN), the BMDCs were injected with LPS (3 mg/one-leg) one day before the infection. The BMDCs were infected with BCG or Mpg or treated with LPS (1 μg/ml) overnight or not infected at all. Then, the BMDCs were incubated with 5 μM of CFSE in PBS for 4 min in a 37 °C water-bath and for 4 min on ice for staining. The prepared BMDCs were resuspended to the same concentration of 10^8^ cells/ml in PBS. The BMDCs in PBS (30 μl) were injected into the right footpads of mice. After 52 hours, pLNs from the injected mice were collected and ground with frosted glass to produce single cells. The single cells were stained with CD11c-APC-cy7. The populations of the migrated BMDCs in pLN were determined by detecting the CD11c ^+^ CFSE-labeled population using flow cytometry (BD LSRFortessa).

### Quantitative real time-PCR (qRT-PCR)

Total RNA from the BMDCs or THP-1 cells infected with BCG or Mpg was extracted using RNA-spin^TM^ Total RNA Extraction kit (iNtRON Biotechnology, Gyeonggi-do, Korea) according to the manufacturer’s protocol. Purified RNA samples were used as templates for qRT-PCR with SensiFAST^TM^ SYBR Lo-ROX One-Step kit (Bioline, Taunton, MA, USA). The primer pairs used to amplify interleukin-10 (IL-10), IL-12, H2DMb (MHC II-associated proteins), CIITA (MHC II transactivator) and CCR7 are given in Supplementary Table 1. The reaction was carried out with an ABI 7500 Real Time PCR System (Applied Biosystems, Foster City, CA, USA) and the data were analyzed using the Ct (cycle threshold) values.

### T cell proliferation assay

To conduct T cell proliferation assay, CD4- and CD8-positive T cells from mice immunized with Mtb (H37Ra) (subcutaneous route at the base of the tail, 1 × 10^6^ CFU in 100 μl of PBS) and BMDCs infected with 10 M.O.I. of BCG or Mpg were used. The schematic schedule for the T cell proliferation assay is described in Supplementary Fig. 2a. Proliferation assays were conducted with fluorescent cytoplasmic tracking dye, carboxyfluorescein diacetate succinimidyl ester (CFSE), CD4 BV421-conjugated anti-CD4 (Clone GK1.5, BD Horizon) and PE-conjugated anti-CD8a (Clone 53-6.7, eBioscience) as described previously^34,38^. The cell cycle profiles were determined using FACS LSRFortessa (BD Biosciences), and analyzed using FlowJo. All the experiments were performed in triplicate.

### Mice and immunization procedures

Female BALB/c and BALB/c_nu (nude) (~25 g, 7-week-old) were purchased from Orient-Bio (Seoul, South Korea) and used for experiments at the age of 8 weeks. The mice were randomly divided into groups with five mice in each group.

For safety test, mice were infected with BCG or Mpg through the tail vein (1 × 10^6^ CFU in 200 μl). At 1, 7, 14 and 28 days after the infection, the mice were euthanized by CO_2_, and their organs (liver, lungs and spleen) were removed for CFU enumeration (Supplementary Fig. 2b).

For CTL analysis, the BALB/c mice were subcutaneously vaccinated twice (with a 2-week-interval) with BCG or Mpg (1 × 10^6^ CFU in 100 μl) at the base of the tail. Two weeks after final vaccination, the vaccinated mice were euthanized, and the spleens were removed for CTL analysis (Supplementary Fig. 2c).

For vaccination test, the BALB/c mice were subcutaneously vaccinated twice (with a 4-week-interval) with BCG or Mpg (1 × 10^6^ CFU in 100 μl) at the base of the tail. The groups were as follows: group I, PBS-vaccinated group (two times); group II, BCG-vaccinated group (two times); group III, BCG- and Mpg-vaccinated group (first vaccination with BCG and second vaccination with Mpg); group IV, Mpg-vaccinated group (two times). Four weeks after the final vaccination, the mice were challenged with Mab or Mtb H37Ra strains via the tail vein (1 × 10^6^ CFU in 200 μl). All the vaccinated mice were euthanized at each time point and their bloods and organs were removed for further immunological study (Supplementary Fig. 2d).

### Ethics Statement

All animal experiments were performed in accordance with institutional guidelines, and the protocol was approved by the Institutional Animal Care and Use Committee (IACUC; approval No. of SNU-IACUC-140711-2) of the Institute of Laboratory Animal Resources at Seoul National University.

### IFN-γ Enzyme-Linked ImmunoSpot (ELISPOT) assay

Splenocytes from the mice immunized with BCG or Mpg were used to conduct ELISPOT assays as described previously^62^. The ELISPOT assay was performed using the mouse IFN-γ (3 μg/ml, clone: AN-18) capture antibody (BD-Biosciences, San Diego, CA, USA) and biotin-labeled mouse IFN-γ (3 μg/ml, clone: XMG1.2) detection antibody (BD-Biosciences) as described previously^38^. Splenocytes (5 × 10^5^ cells/well) were analyzed 24 hour after re-stimulation with whole lysates of Mab or Mtb at a final concentration of 5 μg/ml. The number of spot forming units (SFUs) per well was counted automatically using an ELISPOT reader (AID ELISPOT reader, Strassberg, Germany).

### CFU enumeration assay

Organs (liver, lungs and spleen) from the vaccinated and challenged mice were homogenized with strainers (70 μm mesh) and serially diluted with PBS. The bacterial loads in the organs were determined by plating the homogenized organs onto 7H10 agar plates (supplemented with OADC) for 7 days (in the case of Mab) or 4 weeks (in the case of Mtb) at 37 °C.

### Cytokine determination from mice immunized with *M. bovis* BCG or *M. paragordonae*

Splenocytes from immunized mice were diluted to 1 × 10^6^ cells/well (96 well microplate, 200 μl volume) in RPMI 1640 medium with 10% FBS, and whole lysates of Mab or Mtb strains were added at a concentration of 5 μg/ml for *in vitro* stimulation. The cells were cultured and the supernatants were harvested for IL-2 (BioLegend, San Diego, CA, USA), TNF-α (eBioscience, San Diego, CA, USA), IL-12p40 (eBioscience) (24 hour incubation), IL-10 (R&D Systems, Minneapolis, MN, USA), and IFN-γ (BioLegend) (72 hour incubation) determination by ELISA kit.

### Serum antibody detection

To detect serum antibody ratio, serum samples were collected by cardiac puncture method from the immunized mice after euthanasia with CO_2_. Whole lysates (5 μg/ml) of Mab or Mtb in 0.05 M carbonate-bicarbonate buffer (pH 9.6) were used to coat 96-well plates overnight at 4 °C. Serum antibodies were detected using mouse IgG2a and IgG1 antibodies (BD Biosciences, 1:1,000 dilution) as described previously^38^. The optical density (OD) was determined with a spectrometer at 450 nm^63^.

### Cytotoxic T lymphocyte (CTL) assay

The induced CTL responses were determined as described previously^38,64^. Briefly, the P815 cells (H-2^d^) were transfected with plasmids containing Ag85B-ESAT-6 (pcDNA3.3-Ag85B-ESAT-6) by using Lipofectamine 2000 (Invitrogen, Carlsbad, USA), and then used as the target cells (5 × 10^4^ cells) for the cytotoxic T lymphocyte (CTL) assay. The non-transfected cells were used as negative controls. Splenocytes (5 × 10^6^ cells/well) from the vaccinated mice were co-cultured in RPMI 1640 medium (Biowest, Nuaille, France) with 10% FBS and antigens (Ag85B, 5 μg/ml) at 37 °C in a 5% CO_2_ incubator for six days. These stimulated splenocytes were used as effector cells to evaluate the CTL responses. Cell cytotoxicity was evaluated with lactate dehydrogenase (LDH) in 96-well U bottom plates according to the manufacturer’s protocol (CytoTox 96 Non-Radioactive Cytotoxicity Assay; Promega, Madison, USA) as described previously^38^.

### Hematoxylin and eosin (H&E) staining

For histopathological studies, the lungs were removed from the immunized mice. One fifth of the right lobe of the lung was fixed in 10% formaldehyde (Junsei Chemical Co., Tokyo, Japan) and embedded in paraffin. The paraffin blocks were sectioned at approximately 5 μm thickness and stained with hematoxylin and eosin (H&E).

### Statistical analysis

The data represent the mean ± standard deviation. Student’s *t*-test was used to compare the variance, and the differences were considered statistically significant when the probability values were less than 0.05.

## Electronic supplementary material

Supplementary Information 
