# Supplementary material for: A temperature sensitive Mycobacterium paragordonae induces enhanced protective immune responses against mycobacterial infections in the mouse model
Source: Sci Rep. 2017 Nov 9;7:15230. doi: 10.1038/s41598-017-15458-7 (PMC5680210; doi:10.1038/s41598-017-15458-7)
Supplement: Supplementary file 1 — Supplementary Information [file 41598_2017_15458_MOESM1_ESM.doc]

A temperature sensitive *Mycobacterium paragordonae* induces enhanced protective immune responses against mycobacterial infections in the mouse model

Byoung-Jun Kim, Bo-Ram Kim, Yoon-Hoh Kook and Bum-Joon Kim

Supplementary Figure S1


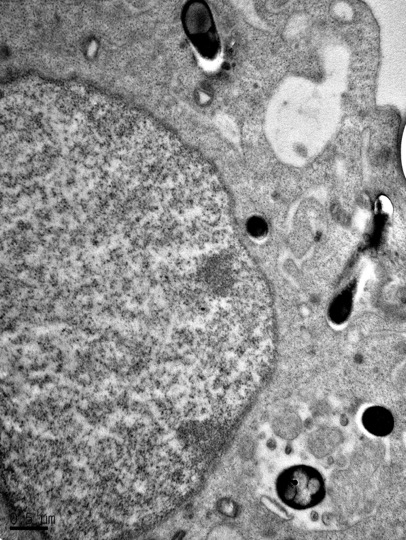

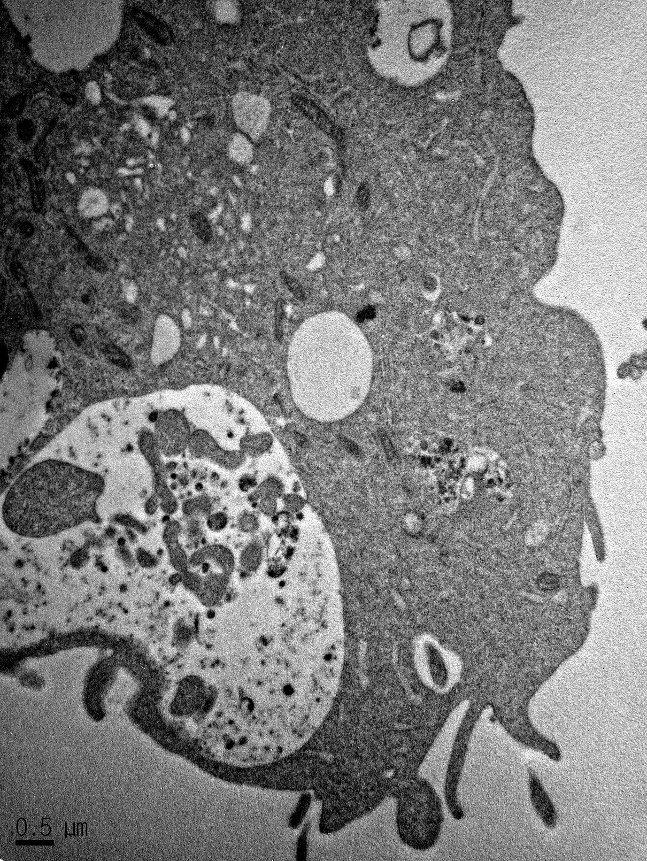


**Transmission electron microscopy (TEM) images of phagocytosed Mpg in J774 cells.** After 24 hours of infection, Mpg was located in the phagosome (left panel). Also, destroyed Mpg was also observed in the phagosome (right panel).

Supplementary Figure S2


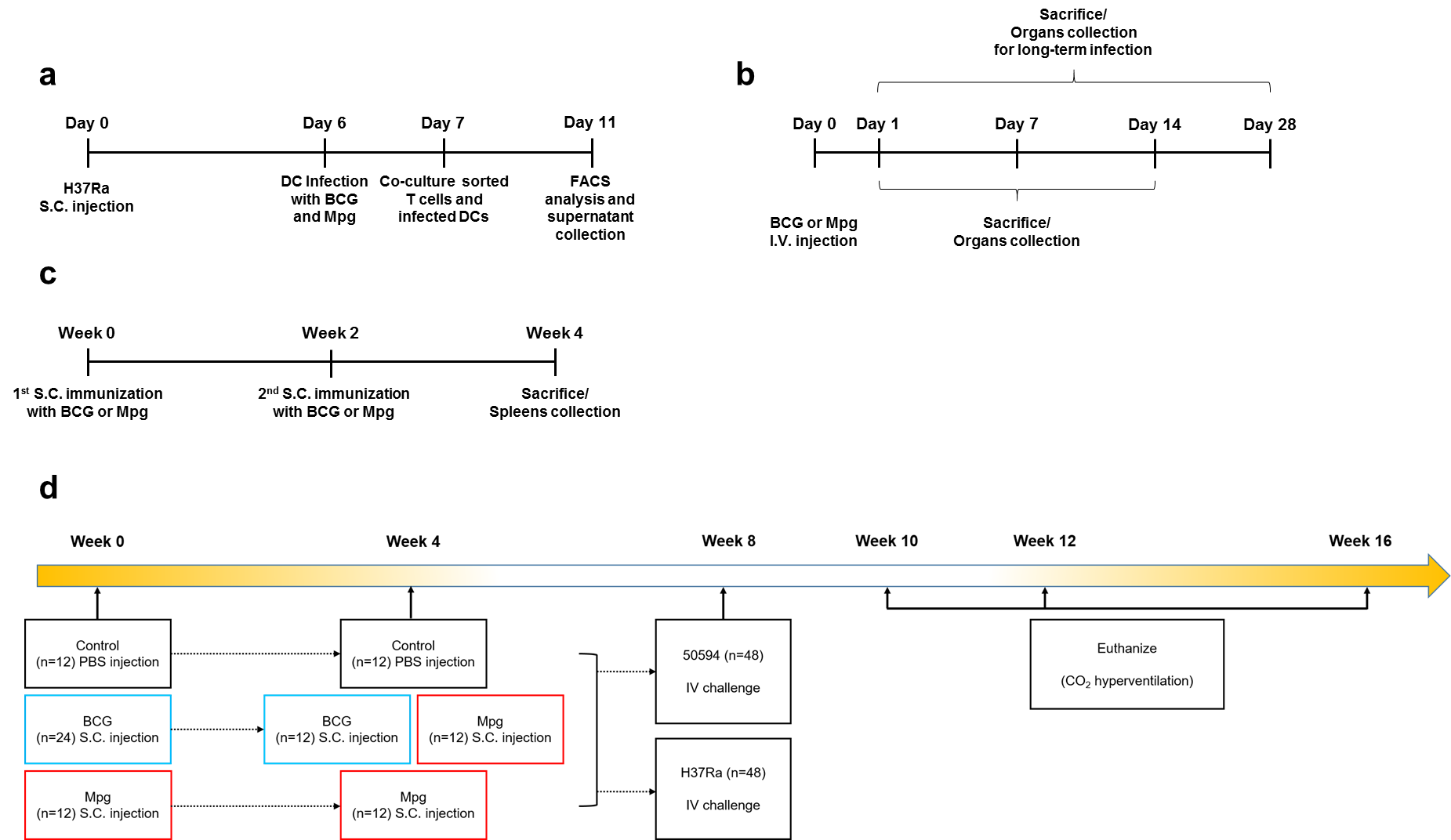


**Schematic immunization schedule for *in vivo* or *ex vivo* tests.** (a) Schematic schedule for BMDC proliferation and T cell proliferation assays. (b) Schematic immunization schedule for safety tests in BALB/c or BALB/c_nu (nude) mice. (c) Schematic immunization schedule for the CTL response assay. (d) Schematic vaccination schedule for examination of the efficacy of Mpg as a potent tuberculosis vaccine.

Supplementary Figure S3

**Evaluation of Mpg persistence in nude mice during long-term infection.** Growth of BCG and Mpg in the organs (lung, liver and spleen) after intravenous inoculation into BALB/c_nu (nude) mice (n = 3 – 4 per group) (* *P* < 0.05, ** *P* < 0.01, *** *P* < 0.001; Student’s t-test).

Supplementary Figure S4

**Comparison of CIITA expression levels between BMDCs infected with BCG and Mpg strains.** The qRT-PCR was used to measure the expression levels of CIITA mRNA in the BMDCs infected with BCG or Mpg. The expression levels represent relative fold changes based on β-actin (* *P* < 0.05, *** *P* < 0.001; Student’s *t*-test).

Supplementary Table S1. Primer sequences for real-time PCR in this study.

| Primers | Sequences (5’ to 3’) |
| --- | --- |
| Mouse β-actin-F | GACGGCCAGGTCATCACTAT |
| Mouse β-actin-R | ATGCCACAGGATTCCATACC |
| Mouse CCR-7-F | TCATTGCCGTGGTGGTAGTCTTCA |
| Mouse CCR-7-R | ATGTTGAGCTGCTTGCTGGTTTCG |
| Mouse CIITA-F | ACGCTTTCTGGCTGGATTAGT |
| Mouse CIITA-R | TCAACGCCAGTCTGACGAAGG |
| Mouse H2DMb-F | CAACAAGGAGAAGACGGCTCA |
| Mouse H2DMb-R | CGCTGTGCTGAACCACG |
| Mouse IL-10-F | ATTTGAATTCCCTGGGTGAGAAG |
| Mouse IL-10-R | CACAGGGGAGAAATCGATGACA |
| Mouse IL-12-F | CCACTGGAACTACACAAGAACG |
| Mouse IL-12-R | GCACAGGGTCATCATCAAAG |
| 18S rRNA-F | AGTCCCTGCCCTTTGTACACA |
| 18S rRNA-R | CGATCCGAGGGCCTCACTA |
| Human IL-10-F | GACTTTAAGGGTTACCTGGGTTG |
| Human IL-10-R | TCACATGCGCCTTGATGTCTG |
| Human IL-12-F | CCTTGCACTTCTGAAGAGATTGA |
| Human IL-12-R | TCCACTGTGCTGGTTTTATCTTT |

Supplementary materials

**Transmission electron microscopy (TEM) analysis**

To visualize the phagocytosed Mpg in macrophage cell line, J774A.1, Mpg was infected with Mpg at 10 M.O.I. for 24 hours as described in Materials and Methods section. Infected cells were washed and fixed for 2 hours in 2% paraformaldehyde and 2.5% glutaraldehyde (in 0.1 M phosphate buffer, pH 7.4) and post-fixed in 1% osmium tetroxide. The fixed samples were dehydrated in ethanol and then embedded in epoxy resin, Epon. The embedded samples were then cut into sections that were stained with uranyl acetate and lead citrate. Samples were visualized using a JEOL JEM1200EX II transmission electron microscope (JEOL, Akishima, Japan)[^1^](#_ENREF_1).

**References**

1 Graham, L. & Orenstein, J. M. Processing tissue and cells for transmission electron microscopy in diagnostic pathology and research*. Nat Prot*o**c** 2, 2439-2450, doi:10.1038/nprot.2007.304 (2007).
